# An ecological analysis of colorectal cancer incidence and mortality: Differences by sexual orientation

**DOI:** 10.1186/1471-2407-11-400

**Published:** 2011-09-21

**Authors:** Ulrike Boehmer, Al Ozonoff, Xiaopeng Miao

**Affiliations:** 1 Department of Community Health Sciences, Boston University School of Public Health, 801 Massachusetts Avenue, Boston, MA 02118, USA; 2 Clinical Research Program, Children's Hospital Boston, 300 Longwood Avenue, Boston, MA 02215, USA; 3 Department of Biostatistics, Boston University School of Public Health, 801 Massachusetts Avenue, Boston, MA 02118, USA

## Abstract

**Background:**

Some have suggested gays and lesbians may carry a greater burden of colorectal cancer. To date, individual sexual orientation data are not available in cancer surveillance registries. This prevents an assessment of differences in colorectal cancer incidence and mortality by sexual orientation, using individual-level data.

**Methods:**

We use an ecological approach to examine differences in colorectal cancer incidence and mortality by county-level sexual orientation data. From the Surveillance, Epidemiology and End Results (SEER) Program we obtain population-based surveillance data on colorectal cancer incidence and mortality from 1996 to 2004. We use Census 2000 data on same-sex partnered households, a proxy of sexual orientation, to derive county-level sexual orientation data. Using multiple regression models, we examined the county-level association of sexual minority density with colorectal cancer incidence and mortality.

**Results:**

After controlling for race and SES, we identify a significant positive association between greater density of sexual minority men and women and colorectal cancer incidence. With respect to colorectal cancer mortality, we identify a positive association with density of sexual minority men, but not women.

**Conclusions:**

In the absence of surveillance data on sexual minority individuals, ecological analyses provide estimates of associations at the aggregate level, thereby providing crucial information for follow-up studies.

## Background

Colorectal cancer (CRC) is the third most common cancer in U.S. men and women. Because sexual orientation data are not included in cancer registries, we do not know the CRC incidence and mortality in sexual minorities, defined as lesbian, gay, and bisexual individuals. We have inconsistent and limited evidence of greater CRC prevalence in sexual minorities. More sexual minority women compared to heterosexual women reported histories of colon cancer in the Women's Health Initiative [1]. Among men, we have limited data on a subpopulation of sexual minority men who are living with HIV infection, indicating that HIV-infected populations presented with a greater prevalence of CRC compared to controls without HIV [2,3].

Prevalence data are clearly inadequate for inferences to CRC incidence and mortality among sexual minorities. However, as others suggested, CRC incidence and mortality may be different by sexual orientation, because of sexual minorities' greater level of risk factors for CRC and their greater access barriers to the health care system, possibly resulting in less timely screening, detection, and treatment of CRC. Life style factors for CRC are a diet that is high in red meats, physical inactivity, obesity, smoking, heavy alcohol use, and type II diabetes. There is sufficient evidence to conclude lesbians have more risk factors due to their higher rates of obesity, smoking, and heavy alcohol use [4-8]. Because of these higher risk factors, the President's Cancer Panel went as far as to suggest "lesbians who use tobacco face risks of breast cancer, colorectal cancer, and other cancers five times higher than those of other women" (page 317) [9]. Gay men's higher level colorectal cancer risk factors are limited to their greater smoking rates compared to heterosexual men [5,6]. Gay men's alcohol use has not been consistently identified as significantly different compared to heterosexual men [7], and gay men are significantly less likely to be overweight or obese compared to heterosexual men [10].

Because of the strong evidence linking CRC screening to reduced CRC incidence and mortality, guidelines recommend screening for CRC for average risk adults at age 50 [11]. So far, data on sexual minorities' CRC screening rates are limited and inconsistent. Studies of CRC screening in women indicate no differences by sexual orientation [12,13], with one study noting that women who ever changed or avoided the facility at which they received screenings because of their sexual orientation were less likely to be adherent to screening guidelines [13]. A recent population-based study of California men concluded that gay and bisexual men have higher screening rates for CRC compared to heterosexual men [14]. A Massachusetts-based study confirmed gay men's higher screening rates, but found no differences in bisexual men's screening rates, compared to heterosexual men [12]. From a study of a predominantly male sample of HIV-infected patients we know that despite HIV-patients' greater health care utilization, they were significantly less likely to have been screened for CRC compared to a control sample without HIV [15], possibly suggesting lower CRC screening among gay men. More complex access issues, such as knowledge about the need for CRC screening, having a usual source of care, receiving a physician recommendation for CRC screening, mistrust of physicians and the health care system [16,17], which have been linked to CRC screening compliance in other underserved populations have yet to be explored among sexual minority populations.

We propose that sexual minorities may carry a greater burden of CRC incidence and mortality due to their greater access barriers to the health care system. For example, previous research has shown that bisexuals have lower health insurance rates [12]; women who have a female partner are less likely to consult medical providers, and less likely to have a usual source of health care which also resulted in greater reports of having unmet medical needs [18]. These various access barriers in combination with higher risk levels among these groups may result in higher CRC incidence and CRC mortality among sexual minorities.

The lack of individual sexual orientation data within cancer surveillance prevent us from testing directly if there is an excess of colorectal cancer incidence and mortality in sexual minority populations. Instead, we turn to ecological analyses to investigate this question at the aggregate level. Ecological analysis means using area-based measures of sexual orientation to assess sexual orientation-related disparities in CRC incidence and mortality because individual-level data on sexual orientation are not available to us. The use of geographic-level data as a substitute for individual level data initially emerged due to a lack of other relevant individual data in cancer surveillance systems, in that cancer registries are not providing individual socioeconomic data, such as income and, education. Several studies used geographic-level socioeconomic data to determine socioeconomic differences in cancer [19-21], after other available individual data such as race or age had been controlled in these models. Our previous work already used ecological analyses to determine area-level differences in breast cancer incidence in counties with higher rates of sexual minority women, defined as women living in female same-sex partnered households according to the Census [22]. The US Census data on same-sex partnered households have been established as a surrogate measure of sexual orientation [23-25]. In the absence of individual-level sexual orientation data and a pressing need to determine cancer disparities by sexual orientation, ecological analyses are an innovative strategy to provide estimates about the existence and magnitude of cancer disparities by sexual orientation. This study, once again relies on Census-derived same-sex partnered households as a proxy for sexual orientation, to examine differences in CRC incidence and mortality by sexual orientation, while stratifying by gender.

## Methods

This is a secondary data analysis of publicly available data of de-identified data. For this reason, the Institutional Review Board deemed this study exempt from protocol review.

### Data sources

#### Census 2000

The decennial US Census conducted in 2000 is an enumeration of the US population. By combining responses from two questions included in the Census, one about the sex of each household member, and the second, about the relationship between each member of the household, which included "unmarried partner" as an answer choice, "same-sex partnered households" are identified [26]. There are some concerns about the same-sex partnered data from the Census, because they only represent coupled sexual minority persons, who are cohabiting and felt comfortable reporting their same-sex partner status [23,24,27,28]. Despite these shortcomings, these data have emerged as a valuable national data source of sexual minorities, because they allow for the determination of where same-sex partnered households are located geographically. In the United States, there are 594,391 same-sex partner households, of which 51% (301,026) are male same-sex partner households [26]. Nationally, the female same-sex partner households consist of a householder with a mean age of 43.4 and a partner with an average age of 42.2 years [26]. Nationally among men, same-sex partner households consist of a householder with a mean age of 44.5 and a partner with a mean age of 42.4 [26]. All Census data files are publicly available. To obtain the aggregate sexual orientation data we rely on the Census Summary File 1 (SF 1) (http://www.census.gov/prod/cen2000/doc/sf1.pdf), which contains information on household relationships asked of all people and housing units. However, this information is not age specific, which is a major disadvantage for our interest in cancer, an age-dependent disease. For this reason, we used also the 5 percent Public Use Microdata Sample (PUMS) of the Census 2000, a stratified random sample of responses to the Census long-form, which contains detailed demographic information, including age, about all members of the household. To obtain estimates on socioeconomic status at the county level, we used the Census Summary File 3 (SF 3) (http://www.census.gov/prod/cen2000/doc/sf3.pdf), which contains detailed information on economic resources, such as vehicles available, value of home, household income, poverty status, as well as variables pertaining to occupation and education from approximately 1 in 6 households. While Census data have a hierarchy of geographical units, the Surveillance, Epidemiology, and End Results (SEER) data are available by county, which limited us to a linkage at the county level.

#### SEER

The Surveillance, Epidemiology, and End Results (SEER) Program is the premier source for cancer statistics in the US. Since 1973, SEER collects data on cancer incidence and survival from various geographic locations throughout the United States. Because SEER expanded its registries gradually from 1973, data are not available from all registries for the same amount of time. Because the sexual orientation data from the Census are from 2000, we wanted nine years of SEER cancer data, from 1996-2004, so that the available Census data in 2000 are the midpoint of our study period. We use data from 12 SEER registries, because these registries have essentially complete data for study years 1996-2004. The 12 selected SEER registries provide us with publicly available cancer data on 215 counties and include diverse regions of the U.S.: Atlanta, Connecticut, Detroit, Hawaii, Iowa, Los Angeles, New Mexico, Rural Georgia, San Francisco-Oakland, San Jose-Monterey, Seattle-Puget Sound, and Utah. According to the SEER website (http://www.seer.cancer.gov), the population covered by SEER is comparable to the general US population with regard to measures of poverty and education. The SEER population tends to be somewhat more urban and has a higher proportion of foreign-born persons than the general US population.

### Measures

Our main independent variable is derived from Census 2000 data on same-sex partnered household, a surrogate for individual sexual orientation data. We aggregate these data at the county-level, expressing either the number of females or males living in a same-sex partnered relationship within a household in relation to the female or male adult population of the county. We call this aggregate variable sexual minority women density (SMWD) or sexual minority men density (SMMD), because it expresses the variation in the density with which resident sexual minority women or men report at the county level. Nationally, there are 293,365 female same-sex partnered households, which means 586,730 women (293,365*2) live in such a household, which after relating it to the national female population (108,133,727), represents a SMWD of 0.54 ((293,365*2/108,133,727)*100). Nationally for men we calculated a SMMD of 0.60 (301,026*2/100,994,367)*100), after relating 301,026 male same-sex partnered households to the male population (100,994,367) [26]. To make these data age specific, we obtained the distribution of sexual minority women, defined as women who live in female same-sex partnered households, across different age groups from the PUMS data, and combined this information with the county-level SMWD. We performed the same calculations for men. Due to the small samples of PUMS at the individual county level, we aggregated PUMS data across the 215 counties in the SEER registry and computed the SWMD or SMMD weight for each age group as: Weight = Number of sexual minority women or men in a specific age group/total number of sexual minority women or men in all age groups.

Our outcomes of interest are sex-specific CRC incidence and mortality, which we obtained from SEER. We limited our sample to men and women aged 18 and older with new, primary diagnoses of CRC and all cases of CRC mortality that were recorded for the years of 1996-2004. There were 61,261 new cases of CRC diagnosed in men and 61,747 in women within the 12 SEER registries. For men, we excluded 15 males under the age of 18 (0.02%), and 3 subjects with an unknown age; for women, we excluded 13 females under the age of 18 (0.02%), and 6 subjects with an unknown age. Over the same years, 1996-2004, in the 12 SEER registries 28,219 CRC deaths were reported in men and 28,313 in women.

To prepare the cancer data for linkage at the county level and to obtain data on covariates we recorded for each woman's and man's case of cancer, age, race, and year of diagnosis, year of death, and county of residence from the SEER. Counts of CRC incidence and mortality were classified into one of 11 age categories, 18-24, 25-29, 30-34, 35-39, 40-44, 45-49, 50-54, 55-59, 60-64, 65-69, and 70 and older, and into one of the three race groups, white, black or other. Other race combines American Indian and Alaska Native, Asian, Native Hawaii and Other Pacific Islander, other race alone, and two or more races. We calculated crude CRC incidence and mortality rates using the total female and male population over 18 years in each county. We also calculated the age-adjusted CRC incidence and mortality rates, which are weighted average of the crude rates in the 11 age groups listed above. The weights were proportions of people in the 11 age groups of the 2000 Standard Million, which is a commonly used standard population for computing age-adjusted incidence or mortality rates [29].

As an additional covariate, we obtained socioeconomic status from the Census 2000 Summary File 3 (SF 3). For each county, we obtained poverty level, defined as the percentage of the population in a county living under the Federal poverty level, which has been found to be the most consistent, easily interpretable variable which accurately measures socioeconomic disparities in health outcomes [21,30].

### Statistical Analysis

Initially, we used descriptive statistics to describe the variation in the independent variables of sexual minority density and the two dependent variables, CRC incidence and mortality by registry. Our primary models examined the county-level association of SMWD or SMMD with age-race-stratified CRC incidence or mortality rates using multivariable Poisson regression models while adjusting for covariates. The models assumed that the age-race-stratified county-level incidence rate (or mortality rate) followed Poisson distributions with conditional mean a function of SMWD/SMMD, race, age group, SEER registry, and socioeconomic status. SAS PROC GENMOD was used to fit the models using counts of age-race-stratified CRC incidence cases (or mortality cases) in each SEER county as the dependent variable. The offset term was the logarithm of the US Census age-race-stratified total adult population in the county. The predictors were SMMD/SMWD, age group, race, SEER registry, and US Census percent in poverty. We evaluated the validity of the assumptions and the goodness-of-fit of the assumed models with residual diagnostic plots and goodness-of-fit statistics such as the Deviance statistic [31]. We also carefully considered other alternative model formulations, including zero-inflated Poisson (ZIP) and negative binomial models. Our selection of Poisson regression models as the final models was based on the goodness-of-fit of the models assessed by the Akaike Information Criterion (AIC) and residual diagnostics plots [32]. We interpreted the estimated regression coefficients from the Poisson models after exponentiation as incidence rate ratios (IRRs) or mortality rate ratios (MRRs), respectively. We used SAS 9.1.3 (SAS Institute Inc, Cary NC) for all analyses.

## Results

Initially we examined the variability of our main independent variable, that is, SMWD and SMMD, by registry and county (results not shown). There is considerable variability by registry with respect to the density of sexual minority women and men in counties. Three registries, Rural Georgia, Hawaii, and Iowa include counties with zero SMWD, meaning within these counties zero percent of the female population live in a same-sex partnered household. The Rural Georgia, Hawaii, New Mexico, and Utah registries have counties with zero SMMD, meaning within these counties zero percent of the male population live in a same-sex partnered household. The counties with the highest mean density of female same-sex households are within the San Francisco-Oakland registry, followed by the New Mexico registry. The counties with the highest density of male same-sex households are within the San Francisco-Oakland registry, followed by the Atlanta registry. Within the geographic areas covered by the 12 SEER registries, the SMWD ranged from 0.00 to 1.44. The mean SMWD for the 215 counties in the 12 SEER registries is 0.43, the same as the average for the 3,141 counties in the US. The SMMD ranged from 0.00 to 2.91. The mean SMWD for the 215 counties in the SEER12 is 0.25, and the mean SMMD for the 3,141 counties in the US is 0.41.

Table 1 presents the accumulated CRC incidence for men and women over nine years from 1996 to 2004 by registry. For men, Iowa has the highest crude incidence rate of CRC, yet after adjusting for age, Connecticut has the highest CRC incidence rate. The results for women mirror those for men, in that again the highest crude CRC incidence rate occurs in Iowa, while after adjusting for age, Connecticut has the highest CRC incidence rate. For men, the average adjusted incidence rate over 9 years for all 12 registries is considerably lower than the US national adjusted incidence rate for 2000. For women, however, the average adjusted colorectal cancer incidence rate for the 12 SEER registries is similar to the national adjusted incidence rate.

**Table 1:** Colorectal Cancer Incidence by SEER Registry from 1996 to 2004

		Incidence in Men			Incidence in Women		
**Registry**	**Counties**	**Incident Cases**	**Crude Incidence Rate**	**Adjusted Incidence Rate**	**Incident Cases**	**Crude** **Incidence Rate**	**Adjusted Incidence Rate**

Rural Georgia	10	250	66.61	68.54	248	58.79	50.72

Hawaii	5	1627	39.61	41.77	1261	30.51	27.91

New Mexico	33	2947	51.56	56.39	2603	42.82	41.62

Utah	29	2347	34.82	48.43	2261	32.83	39.48

San-Jose Monterey	4	3268	40.23	55.20	3080	38.79	42.33

Atlanta	5	3532	37.40	60.26	3759	37.74	49.30

Iowa	99	6663	69.92	70.71	6923	67.84	54.32

Connecticut	8	7539	68.76	71.99	7852	64.83	55.32

Detroit	3	7152	56.24	63.86	7703	54.78	50.80

Seattle	13	6122	45.28	54.71	5952	42.81	43.55

San-Francisco-Oakland	5	6927	49.27	59.45	6800	46.35	45.48

Los Angeles	1	12869	42.85	57.60	13286	42.01	45.89

All 12 Registries	215	61243	48.87	59.91	61728	46.76	46.99

2000 U.S. (from CDC)*	3,141	17,389	57.0	66.6	17,662	53.6	47.9

*U.S. Cancer Statistics Working Group. *United States Cancer Statistics: 1999-2007 Incidence and Mortality Web-based Report*. Atlanta: U.S. Department of Health and Human Services, Centers for Disease Control and Prevention and National Cancer Institute; 2010. Available at: http://www.cdc.gov/uscs.

In Table 2 we present the equivalent information for CRC mortality by registry. Of the 12 SEER registries, Iowa has the highest CRC mortality rate for men and women. Iowa retains the rank as the registry with highest CRC mortality for both men and women, even after adjusting for age. The 12 SEER registries combined have a higher incidence rate of CRC mortality in men and women than the national US mortality rate for men and women.

**Table 2:** Colorectal Cancer Mortality by SEER Registry from 1996 to 2004

	Mortality in Men				Mortality in Women		
**Registry**	**Counties**	**Incident Deaths**	**Crude Mortality Rate**	**Adjusted Mortality Rate**	**Incident Deaths**	**Crude Mortality Rate**	**Adjusted Mortality Rate**

Rural Georgia	10	116	30.90	32.28	111	26.31	22.58

Hawaii	5	409	9.96	10.62	276	6.68	6.02

New Mexico	33	1395	24.41	27.29	1207	19.97	19.44

Utah	29	1054	15.79	22.81	1019	14.87	17.97

San-Jose Monterey	4	1328	16.35	23.23	1275	16.06	17.55

Atlanta	5	1468	15.54	26.96	1606	16.12	21.61

Iowa	99	3360	35.26	35.75	3576	35.04	27.50

Connecticut	8	3259	29.72	31.44	3402	28.09	23.64

Detroit	3	3736	29.38	34.02	3835	27.27	25.13

Seattle	13	2982	22.05	27.55	2882	20.73	21.11

San-Francisco-Oakland	5	3241	23.05	28.48	3265	22.26	21.76

Los Angeles	1	5871	19.55	26.91	5859	18.53	20.23

All 12 Registries	215	28219	22.53	28.28	28313	21.46	21.46

2000 U.S. (from CDC)*	3,141	28,484	20.6	25.0	28,950	20.1	17.5

*U.S. Cancer Statistics Working Group. *United States Cancer Statistics: 1999-2007 Incidence and Mortality Web-based Report*. Atlanta: U.S. Department of Health and Human Services, Centers for Disease Control and Prevention and National Cancer Institute; 2010. Available at: http://www.cdc.gov/uscs.

In Table 3 we show the multiple regression results for colorectal cancer incidence in the male and female population, with each model, adjusted for age, registry, race, and poverty level. The model for the male population shows a positive significant relationship between SMMD and county-level CRC incidence rate. The incidence rate ratio of 1.04 indicates that with each one-unit increase in a county's SMMD, the CRC incidence rate in men increases by 4%. The relationship between the SMWD and CRC incidence is a significant positive relationship as well; a one-unit increase in a county's SMWD is associated with a 6% increase in a county's CRC incidence rate. In both models for men and women, a county's poverty level has no significant association with CRC incidence, whereas the association between race and CRC incidence is significant. Among both men and women, the association between black race and CRC incidence is positive, indicating that counties with more Black men and women have more CRC cases compared to white men and women. Other race compared to white race has an inverse relationship with CRC incidence, in men and women, indicating fewer CRC cases in counties with other race populations compared to white populations.

**Table 3:** Multiple Regression Model for Colorectal Cancer Incidence in the Male and Female Population*

	Male Population					Female Population				
**Parameter**	**Estimate**	**IRR**	**95%CI**		**p-value**	**Estimate**	**IRR**	**95%CI**		**p-value**

SMD	0.0391	1.0399	1.0203	1.0599	< 0.0001	0.0609	1.0628	1.0068	1.1218	< 0.0001

Poverty Level	0.0009	1.0009	0.9982	1.0037	0.5061	0.0019	1.0019	0.9992	1.0046	0.1767

Black vs. White	0.1286	1.1372	1.1047	1.1707	< 0.0001	0.2418	1.2736	1.2396	1.3085	< 0.0001

Other vs. White	-0.5135	0.5984	0.5817	0.6156	< 0.0001	-0.5536	0.5749	0.5581	0.5922	< 0.0001

*results also adjusted for age and registry

In Table 4 we present the regression models for CRC mortality, with each model for the male and female population adjusted for the covariates, age, registry, race, and socioeconomic status. For men, we find a significant positive association between SMMD and CRC mortality, in that a one-unit increase in a county's SMMD increases the county's CRC mortality by 4%. Poverty level is also significantly associated with CRC mortality. Among men, black race compared to white race has a significant positive association with CRC mortality, suggesting that counties with more black men have higher CRC mortality. Counties with more other race men compared to white men have a significant inverse relationship with CRC mortality. Among women, the association between SMWD and CRC mortality is not significant. A county's poverty level is significantly associated with CRC mortality. Among women, black race is significantly positive associated with CRC mortality, indicating that counties with more black than white women have greater CRC mortality. Counties with more other races have significantly fewer CRC deaths compared to counties with more white women.

**Table 4:** Multiple Regression Model for Colorectal Cancer Mortality in the Male and Female Population*

	Male Population					Female Population				
**Parameter**	**Estimate**	**MRR**	**95%CI**		**p-value**	**Estimate**	**MRR**	**95%CI**		**p-value**

SMD	0.0428	1.0438	1.0118	1.0767	0.0069	0.0539	1.0554	0.9681	1.1506	0.2210

Poverty Level	0.0118	1.0119	1.0079	1.0159	< 0.0001	0.0068	1.0068	1.0029	1.0108	0.0007

Black vs. White	0.2698	1.3098	1.2581	1.3636	< 0.0001	0.3052	1.3570	1.3052	1.4107	< 0.0001

Other vs. White	-0.7933	0.4523	0.4312	0.4745	< 0.0001	-0.8934	0.4093	0.3887	0.4310	< 0.0001

*results also adjusted for age and registry

## Discussion

Using ecological analyses, we have shown that counties with greater sexual minority density tend to have a higher incidence of CRC. These significant positive associations between sexual minority density and CRC incidence at the county-level have been fully adjusted for age, registry, race, and socioeconomic status. Our results show that after adjusting, an increase in a county's sexual minority men density increases CRC incidence by 4%, whereas the comparable increase in sexual minority women density relates to an increase of 6%. The results for CRC mortality differ by gender. There is a significant positive association between county-level density of sexual minority men and CRC mortality, while there is no significant association between sexual minority women density and CRC mortality.

To our knowledge this is the first study to identify associations between CRC incidence and mortality and sexual minorities, in that we found greater CRC mortality rates in counties with more households of sexual minority men, and more CRC incidence with greater density of sexual minority women and men. Previously reported associations between sexual minorities and CRC have been limited to higher CRC risk factors among lesbians [4-9] or greater smoking rates among gay men [5,6]. With respect to the latter finding, earlier studies pointed to higher CRC rates in HIV-infected populations [2,3]. Thus higher CRC mortality in areas with more sexual minority men might be explained by comorbid HIV-infection as suggested by a recent study that indicated greater HIV-related mortality among sexual minority men [33]. No previous research has established a link between CRC incidence, mortality and sexual orientation, although such a link is plausible given the established risk factors and barriers to access. Our study is one of the first to examine this association empirically.

Other aspects of our findings, such as the racial disparity in colorectal cancer incidence and the SES and racial disparities in colorectal cancer mortality, are consistent with prior individual-level and aggregate-level research, in that Black men and women have a higher CRC incidence and mortality compared to White men and women [34,35] as identified by our ecological analyses. Colorectal cancer incidence and mortality have also been shown to have a complex relationship with race and socioeconomic status, in that some studies found racial disparities were reduced or even explained when socioeconomic status was measured [36,37]. In our study, the ecological analyses of CRC incidence did not show an association of poverty and incidence, yet when focusing on mortality both race and poverty were significantly associated with mortality. We used poverty level to address socioeconomic disadvantage, another ecological study used educational attainment rather than poverty and linked it to CRC outcomes [38].

The ecological nature of our study is an important caveat, in that we used an area-based indicator of sexual minority status because individual data are not available. We see this in the same realm as earlier ecological analyses that focused on racial or socioeconomic differences in health, while relying on geographic-area indicators such as SES or percent of Blacks in a county. These earlier studies with respect to race or SES have been able to show that area-based measures are strongly and independently associated with risk factors, access to healthcare, and health outcomes, sometimes even when individual measures of socioeconomic status are considered [39]. We hope that with respect to sexual minority status, other research will seek to substantiate the link between sexual orientation and CRC identified by this study.

This study has several limitations. We cannot incorporate the complexity of other ecological analyses, because individual-level sexual orientation data are not available to supplement our models. In addition, CRC incidence and mortality data are not available to us at geographic scales smaller than the county level. We recognize that using census tract or block level rather than the county as the unit of analysis may yield different results. This has been well documented with respect to SES data, in that smaller geographic units such as the census tract provide more consistent SES gradient compared to larger geographic units [40]. Future research that focuses on sexual orientation will need to provide similar formative work to determine an appropriate geographic scale for studies of disparities linked to sexual orientation. In recognition of the limitations imposed by the ecological approach, we caution that the inference is at the level of the county, and not at the level of the individual. To conduct these analyses, we used a proxy for sexual orientation, same-sex partnered households. This proxy measure is known to be an undercount of the sexual minority population. Specifically, single sexual minority individuals, those who are partnered yet not residing with their partner, and individuals who do not feel comfortable disclosing a household member as their unmarried partner [23] are not captured by this proxy measure. By controlling for poverty and race, both well-known contributing factors to CRC incidence and mortality, we suggest the observed associations between sexual minority density and CRC incidence and mortality are robust.

## Conclusions

We consider this approach novel and suitable for identifying sexual orientation disparities, which we then suggest should be subject to further investigations. Our estimates of associations at the aggregate level shall be the starting point for further research to examine and better understand differences in CRC incidence and mortality by sexual orientation. Our results are consistent with our previous analysis, which identified breast cancer disparities due to sexual orientation [22]. If ecological analyses can be validated by future studies as a valid tool for identifying differences with respect to sexual orientation, we may apply this methodology to monitor the health of sexual minorities, which we cannot do with presently available surveillance tools.

## Conflict of interest

None of the authors has a conflict of interest.

## Authors' contributions

UB originated the study, led the writing, and interpreted the findings. AO provided his statistical expertise to the analysis and helped to interpret the findings. XM conducted the analyses and helped to interpret the findings. All authors contributed in significant ways to the final article by reviewing and discussing earlier drafts. All authors read and approved the final manuscript.

## Pre-publication history

The pre-publication history for this paper can be accessed here:

http://www.biomedcentral.com/1471-2407/11/400/prepub
